# Selecting optimal wearables for measuring physiological arousal in robot-delivered mindfulness-based exercises

**DOI:** 10.1080/01691864.2024.2369797

**Published:** 2024-07-11

**Authors:** Stefania V. Vacaru, Lok-Pui Lau, Kyra Frederiks, Paula S. Sterkenburg, Emilia Barakova

**Affiliations:** a Department of Clinical Child and Family Studies & Amsterdam Public Health, Vrije Universiteit Amsterdam, Amsterdam, Netherlands; b Department of Psychology, New York University – Abu Dhabi, Abu Dhabi, United Arab Emirates; c Department of Industrial Design, Eindhoven University of Technology, Eindhoven, Netherlands

**Keywords:** Electrodermal activity, robot-delivered intervention, empatica E4, shimmer device, wearables

## Abstract

While social robots show promise for therapeutic interventions, accurate assessments of (vulnerable) participants' affective outcomes require attention. The careful selection of devices for recording autonomic processes in response to stress-inducing and relaxing exercises is essential to ensure data quality recordings and participants' comfort. This foundational study assessed two commonly utilized devices to record electrodermal activity (EDA), indexed through skin conductance, concerning their sensitivity to stress-relaxation manipulations and social validity during a robot intervention: a sock with a Shimmer device and a wrist-worn Empatica E4. We aimed to select the most sensitive and easy-to-wear one as a precursor to a larger intervention study featuring mindfulness-based relaxation exercises delivered by an NAO robot. The findings, based on 28 healthy Dutch-speaking adult volunteers wearing both devices, revealed sensitivity in detecting EDA variations in arousal following stressful (increase) and Robot-delivered mindfulness-based relaxation (decrease) exercises, further corroborated by self-reports. Bland-Altman results suggested little agreement between the two devices and lower sensitivity for the Empatica E4. No statistically significant differences concerning wearing comfort between the Empatica E4 and the Shimmer devices emerged. Although both devices independently showed sensitivity to stress/relaxation manipulation, the choice for one or the other should be informed by the activities in the intervention.

## GRAPHICAL ABSTRACT

**Figure:**
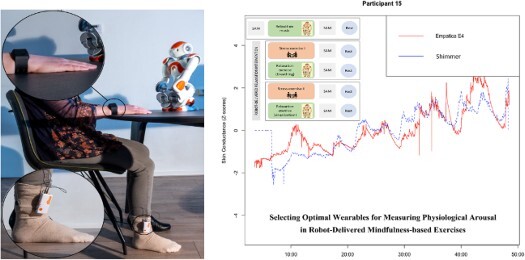

Socially assistive Robots (SARs) are emerging as an important therapeutic tool, offering unique advantages in supporting individuals with various needs [1,2]. Budding evidence testifies to humanoid robots’ positive contribution to psychological well-being as an intervention tool for individuals with or without disabilities across ages [3–6]. The interactive nature of SARs and consistent and non-judgmental demeanor offer a supportive environment that can enhance the therapeutic process [7,8]. They can be programed to deliver tailored therapeutic activities, monitor progress, and provide feedback, thereby supporting patients and healthcare professionals [9]. Moreover, SARs offer companionship and emotional support [10], which promotes self-disclosure in clients with disabilities [11], a crucial therapeutic ingredient. Integrating SARs in therapeutic contexts represents a building block in blending technology with personalized care, potentially transforming how we design and deliver rehabilitation and mental health support [12].

Interestingly, most outcomes of Robot-delivered interventions rely on self-reported psychological well-being [3] or behavioral assessments, neglecting implicit physiological indices. Some evidence suggests that in hospitalized children, SARs reduced physiological arousal and increased positive affect [13]. Similarly, people with dementia showed more positive engagement matched by physiological profiles during an interaction with a social Robot [14]. These studies provide initial evidence of the invaluable benefit of physiological recordings in complementing behavioral assessments, especially for populations with difficulties detecting and/or reporting their inner affective states. Yet, our understanding of optimally detecting physiological stress in vulnerable populations while balancing data accuracy, richness, and individuals’ comfort is lacking.

The body orchestrates the parasympathetic (PNS) and the sympathetic (SNS) systems, which act rapidly in a dynamic equilibrium to induce/reduce alertness and arousal, which creates an optimal self-regulatory stress response. The SNS and PNS innervate the cardiac muscle, whose function can be seen as the accelerator and the brake pedals of cardiac activity, and regulate electrodermal activity (EDA) through the sweat glands. EDA is a crucial physiological marker in understanding stress regulation and the autonomic nervous system [15]. It offers insights into autonomic changes in the electrical properties of the skin, independent of the parasympathetic activity. This characteristic of EDA makes it an invaluable tool in complementing behavioral data, particularly in stress and emotional processing research [16]. While behavioral observations capture external manifestations of emotion and cognition, EDA taps into the SNS's subtle, internal reactions that are not always overt. Skin conductance (SC), a marker of EDA, is connected to emotional arousal, whether due to stress, excitement, fear, or other intense emotions [17]. Given the ability of this measure to capture subtle changes in the autonomic system, it represents an important assessment tool in psychological and experimental research, especially in subjects whose ability to articulate emotional states may be impaired or in fields lacking appropriate assessment tools. Furthermore, individual differences in emotional experiences are likely more nuanced than a self-report questionnaire can capture, but an implicit physiological index may accurately do so. Furthermore, previous studies have shown that overt behaviors do not always match with covert physiological processes. Particularly, the converging findings of these two studies show that behavioral minimization may be related to higher arousal, indexed by increases in electrodermal activity under a stressful task of recollecting early caregiving experiences. Accordingly, to accurately capture emotion regulation processes and arousal, particularly in participants who may not be able to verbalize subjective affective states (e.g. infants and individuals with disabilities), implicit measures of the autonomic system are imperative.

Combining psychological and physiological assessments is essential for interventions targeting stress reduction in vulnerable individuals, for instance, with intellectual or physical disabilities. To exemplify, Van Wingerden and colleagues [10] tested an NAO Robot, a programable humanoid Robot, to teach emotion regulation skills to reduce worry in individuals with an intellectual disability (ID). While there is merit in such an empirical endeavor, one widely reported limitation lies in assessing the outcomes, which relied solely on self-report questionnaires. The field of disability research lacks reliable and well-validated instruments for evaluating critical inner self-regulation processes in individuals with ID. In the abovementioned study, the reduction in worry following the intervention did not surface statistically. Yet, the participants and their caregivers reported positive feelings, which did not match the questionnaire scores. This contradiction between the collected data via available questionnaires and the anecdotal reports begs whether the intervention might have been ineffective in reducing worry or if the researchers lacked the tools to detect a meaningful effect. Particularly for individuals with ID, it may be difficult to express their feelings, primarily based on a questionnaire that may pose additional language comprehension barriers [18]. We advance the proposition to complement psychological measures with physiological stress signatures to address such limitations. Aligning measures to obtain accurate insights into the potential of interventions is imperative to inform the design of cost-effective interventions and prevent clients’ burden and financial losses associated with possibly erroneously discarding an intervention that may not have been accurately detected.

This endeavor does not come without challenges. Recent technological and biomedical advances saw the development of many wearables varying in design, anatomical sites, accuracy, and possibly comfort. Considerations regarding wearability comfort for real-time recording of physiological stress markers are essential as they may interfere with individuals’ autonomic reactions, independent of the intervention effects. Accordingly, using non-invasive and ergonomic equipment is pivotal to preventing anxiety in participants, also commonly known as the white-coat effect, or anxiety for medical staff and/or simply being aware of being assessed [19]. The wrist and the foot are two common anatomical recording locations for electrodermal activity [20], which can be assessed via different recording devices varying in type of sensors or customization. For instance, Shimmer devices are tailored to research and clinical applications by offering an extensive range of materials and flexibility in the customization for specific research goals and populations [21]. One particular implementation of the Shimmer device was via a sock. The sensors are placed in the sole of a sock, which has been developed for clients with disabilities [22,23]. As such, wearing the device goes unnoticed, preventing the clients from being distracted and possibly nervous about it. In turn, wrist-worn devices are more readily available and are tailored to customer and fitness use, no different from wearing a watch, which most individuals tolerate for prolonged use. Accordingly, these devices have become an attractive recording alternative to the hands. Nevertheless, these devices have shown mixed findings compared to other EDA recording devices. They are thought to trade off the level of precision for higher comfort, because a high level of sweat is needed to be picked by the dry electrodes integrated in the device [15,24]. However, balancing accuracy and comfort is vital in experimental psychophysiological research with vulnerable populations. Following these considerations, our study sought to test a sock-integrated Shimmer device and a wrist-worn Empatica E4 device since these mirror daily worn items, which may prevent the participants from being distracted by them.

In sum, unprecedented technological advances in Robotics and biomedical fields offer new venues for improving the quality of life of individuals with various needs at all ages by designing appropriate interventions [25–28]. Yet, integrating different devices to detect Robot-delivered intervention effects is still in its infancy, especially for vulnerable individuals who may benefit the most from subtle online monitoring of stress markers. The goal of this study is twofold. First, we aim to assess two types of wearables for monitoring real-time arousal: a sock with a Shimmer device integration developed in our lab [29,30; https://www.bioresponsesandcare.nl/onderzoek.html] and the Empatica E4 wrist-worn band [31–34], concerning data accuracy and comfort wearability. The second goal is to test physiological stress reduction during a Robot-delivered intervention, based on Van Wingerden and colleagues [10], as evidenced by electrodermal activity responses and self-report. The choice of employing an NAO robot for intervention delivery was dictated by our ongoing research line employing AI-informed technologies for clients with disabilities (https://osf.io/g7r59), making this study a foundational step to establish the Robot-delivered intervention feasible and the electrodermal measuring devices sensitive to capture physiological changes as part of the experimental manipulations. We hypothesize that *H1)* the sock-integrated Shimmer device will not differ from the wrist-worn Empatica E4 band regarding the signal or wearing comfort. Next, we hypothesize *H2)* that an NAO Robot-delivered intervention will lead to stress reduction indexed a decrease in electrodermal activity (i.e. skin conductance, henceforth SC), as assessed by two recording devices and self-reporting following a lab-based stress-eliciting math exercise.

Based on the novelty of this research, which aims to test the feasibility of integrating different technologies for psychobiological stress reduction, the study will test a sample of typically developed individuals. These foundational findings will be leveraged in future clinical and experimental work with individuals with disabilities.

## 1. Methods

### 1.1. Participants

Twenty-eight healthy Dutch-speaking adults (*M_age _*=23.68, *SD *= 3.65; 14 females) were included in the study. They were recruited through snowball sampling with the following inclusion criteria: 18–30 years and minimum Dutch language level B1. The exclusion criteria were having a diagnosed psychological or physical condition (e.g. depression, visual disability). An online screening questionnaire was used for the preselection. Three volunteers were precluded from participation due to a pre-existing psychological diagnosis. At the start of the experimental procedure, participants filled in the Four-Dimensional Symptom Questionnaire (4DSQ) [35], yet no additional participants were excluded from the final sample. Demographic characteristics are summarized in Table 1. Recruitment strategies included word of mouth and snowballing at the Saxion Deventer University of Applied Sciences in The Netherlands (Nov 2021 – Jan 2022). The study was approved by the Scientific and Ethical Review Board (VCWE) of the Faculty of Behavior & Movement Sciences, Vrije Universiteit Amsterdam (VCWE-2022-051), and the Medical Ethical Committee of the Vrije Universiteit Medical Center Amsterdam (Niet-WMO 2022.0175). Before visiting the lab, participants received an information letter and the consent form explaining the experimental procedure via email. During the lab visit, participants provided written consent to participate in the study following the Declaration of Helsinki. Data collection occurred in a designated lab room at the Saxion Deventer University of Applied Sciences and at the Bartiméus Institute, Deventer, The Netherlands. At the end of the visit, they were debriefed and compensated with two gifts, which were approximately 10 euros.

**Table 1.** Descriptive results of the sample and variables in the study (N = 28).

	%	M (SD)	Min-Max
Sex assigned at birth	50% females		
Age		23.68 (3.65)	18–30
Education level	29% of secondary vocational education		
Social validity	71% higher education/university		
Satisfaction experimenter		21.96 (4.48)	14–30
Satisfaction Robot		33.78 (2.14)	28–35
Wearing comfort Empatica E4		25.92 (3.14)	20–30
Wearing comfort sock-integrated Shimmer		26 (4.11)	15–30

Notes. % percentages, M = mean, SD = standard deviation, Min = minimum, Max = maximum

### 1.2. Procedure

**Figure 1.**
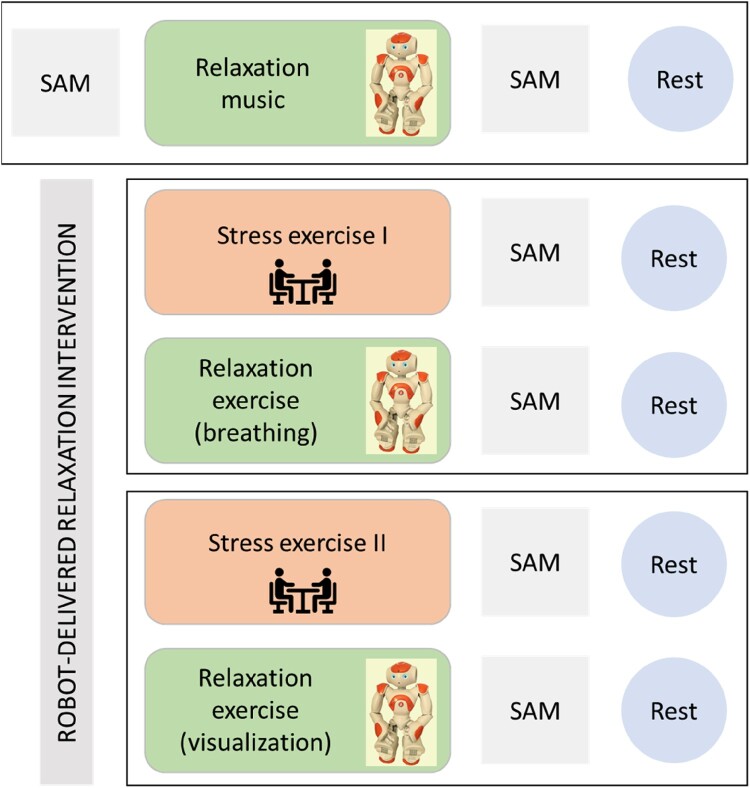
Study design and order of exercises in the intervention. SAM = Self-assessment Manikin; Rest = 2 minutes break.

This is an experimental study using a within-subjects design. The study comprised five exercises: 3 relaxation-oriented and two stress-eliciting. Participants were notified not to consume caffeine or other intoxicating substances or exercise 1.5 hours before the session, as these activities may interfere with the physiological assessments. After the participants entered the lab room, they were seated at a table, and the experimenter explained the procedures of the experiment. The experimenter then asked the participants if they had any questions and, if not, to sign the informed consent. Participants were informed that they could interrupt their participation at any time without consequence. Next, the experimenter applied the wearables to the participant and set up the Robot on the table at approximately 50 cm from the participant. The first part started with the participants filling out a pictorial assessment of arousal and valence of their emotional state Self-Assessment Manikin; SAM [36]. Afterward, the Robot introduced itself and offered to play a relaxing song via a headset for approximately 3.37 minutes, followed by another SAM assessment and 2 minutes of rest. Next, the experiment consisted of two similar parts that only differed in the type of exercises. Both parts unfolded: a stress-eliciting exercise conducted with the experimenter and a relaxation exercise delivered by the Robot. After each exercise, a SAM was completed, and a 2-minute rest period was taken (Figure 1, study design). The researcher would sit out of sight during the relaxation exercises and rest periods. A social validity questionnaire was filled out at the end of the experiment. Physiological recordings of skin conductance were taken throughout the whole session. Finally, the experimenter removed the wearables, debriefed, and thanked the participants. The entire procedure lasted approximately one hour.

### 1.3. Mindfulness-based relaxation intervention

The relaxation intervention, rooted in mindfulness-based practices, comprised five exercises in a fixed order. The first exercise (baseline) consisted of listening to a song (Gymnopédie No.1 by Erik Satie) via a headset for 3.37 minutes. Participants were able to adjust the volume. In the experimental phase, two stress-eliciting exercises delivered by the experimenter would alternate two relaxation exercises delivered by the Robot. These exercises were adaptations of traditional mindfulness techniques, specifically tailored by an experienced therapist skilled in providing compassion and mindfulness training to individuals with visual and intellectual disabilities [10,25,37,38].

In the first stress-eliciting exercise, the researcher followed the instructions of the Wechsler Adult Intelligence IV-NL (WAIS-IV-NL) numerical series subtest. Although this test consists of three sections (forward, backward, and sort), two sections (backward and sort) were conducted. Each section consists of eight items (excluding the practice item), and each item has two numerical series. The number of the first item consists of 2 numbers; in the following item, a number is added to the series. The series in item 8 would consist of 9 numbers. In the backward section, the participants had to recite the numbers backward, and in the sort section, the participants had to order the numbers from small to large. The WAIS-IV-NL manual was followed: 1) when two series of the same items were said incorrectly, the section was done, and 2) the researcher read the series aloud only once. A rule was added: the researcher asked for an answer if the participant didn't answer within 15/20 s to heighten the intensity. Next, a relaxing exercise was conducted, and the robot guided a breathing exercise for 3.5 minutes. During the exercise, the Robot made movements corresponding to its speech, such as putting its hands on its stomach while breathing, requiring the participant to mirror them.

In the second stress-eliciting exercise, the participant had to subtract 13 from 1074 as fast as possible within 3 minutes. The researcher would call out if a mistake were made. The participant could only proceed when the correct answer was listed. If the participant wanted to give up within the timeframe, the researcher would encourage them to keep trying. Finally, a second relaxing exercise lasting 3.5 minutes was conducted. The Robot performed a visualization exercise in which the participants were guided through the woods and ended up at a waterfall where they could wash off their worries. The order of the stress-inducing and relaxation exercises was fixed.

### 1.4. Apparatus

**Figure 2.**
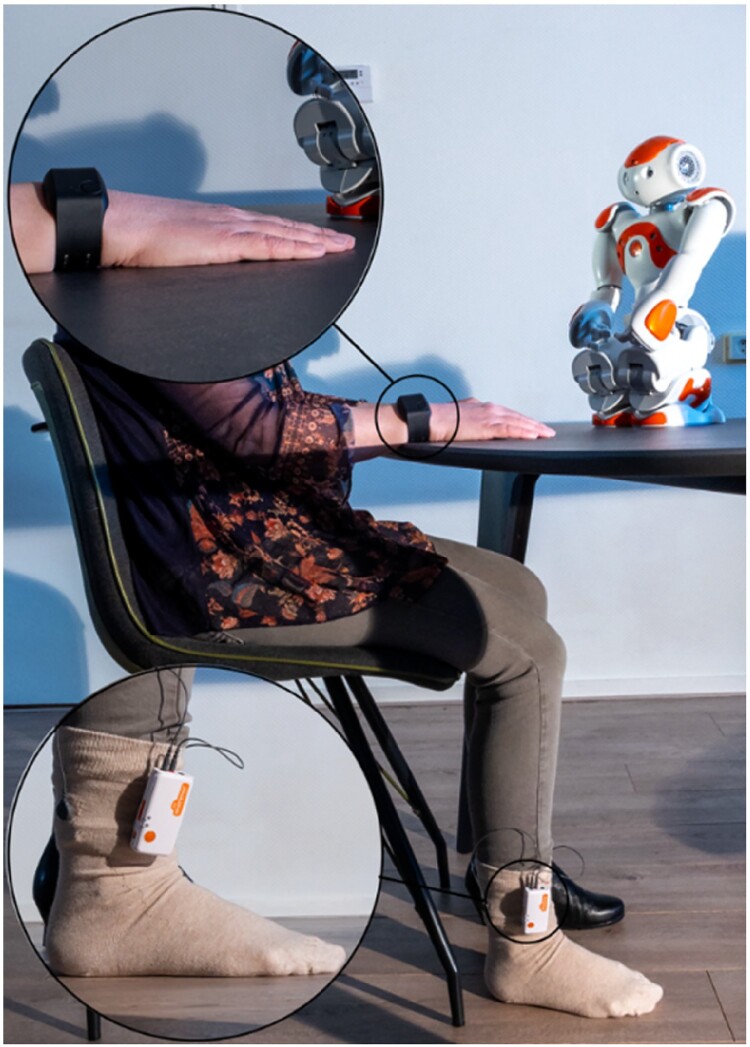
Illustration of the setup and equipment featuring the NAO Robot, The sock-integrated Shimmer device, and the Empatica E4.

*The NAO Robot* (Aldebaran Robotics, France) [39,40] (Figure 2) can move its head, arms, hands, fingers, legs, and feet, and it has four embedded speakers. The Robot's movements, speech, and interaction sequences for this project were programed by an expert from TiViPE (Helmond, The Netherlands). The speech's tone and speed were altered based on feedback from co-researchers with mild to moderate intellectual disabilities. During the exercises, the Robot moved its legs, fingers, or arms and distributed its weight from left to right and back to make the interaction more natural. The Robot's voice and speech were enhanced by adjusting the speed (e.g. expressing excitement), volume, pauses, and tempo. In this study, we used a female voice. The humanized voice was programed and is available via https://www.tivipe.com.

*Empatica E4 bracelet* [32] is a wrist-worn wireless device that measures physiological signals, including electrodermal activity, through a sensor touching the skin. Previous studies have demonstrated the validity and reliability of the E4 in measuring physiological signals in various populations and settings [31,33,41]. The Empatica E4 consists of two sensors located in the wristband strap and allows data acquisition at 4 Hz. In this study, we extracted electrodermal activity or skin conductance, measured in µS.

*The Shimmer^TM^3 GSR + unit* was connected to a sock, recording skin conductance via two fabric sensors on the inside sole. The Eindhoven University of Technology, the Vrije Universiteit Amsterdam, and Bartiméus developed the sock wearable [38,42]. With two embedded textile electrodes on the sole, this sock is a non-invasive, wireless, and wearable tool to record EDA [30,43] (see Figure 2). The Shimmer was worn with an anklet around the ankle connected to the sock, and a 6A current was sent from one fabric sensor to the other. Before recording, the calibration was performed using the Shimmer3 Calibration Stand. The Shimmer^TM^ unit used Bluetooth to connect to an existing mobile app, where the data were collected, and a text file was created per participant. The Shimmer^TM^ unit has a sampling frequency of 50 points per second (50 Hertz) and gathers raw and standardized skin conductance and resistance measures. The sock-integrated Shimmer device gives insight into emotional arousal and stress levels by converting the raw skin resistance data to skin conductance (1000/1 kΩ = 1 µS).

### 1.5. Instruments

*The Self-Assessment Manikin (SAM* [36]) *assessed behavioral arousal and valence*. SAM is a nonverbal pictorial technique featuring a black-and-white cartoon-like character representing different levels of these emotional dimensions. It allows participants to easily indicate their affective states along valence (unpleasant-pleasant) and arousal (calm-tensed). Participants are asked to rate their emotional state by selecting a manikin from each scale that best corresponds to their feelings. Scores ranged from 1-9, with lower scores indicating more unpleasantness/calm and high scores indicating more pleasantness/tension.

*Social validity* was assessed with a questionnaire of 28 items, based on the System Usability Scale, in which the respondents were asked to rate their level of agreement with each statement on a 5-point scale ranging from ‘1 = completely disagree’ to ‘5 = completely agree’. The questionnaire includes items related to the respondents’ experience with the experimenter and Robot Bart, including their enjoyment, understanding, and comfort level during the interactions. Moreover, during the study, the questionnaire assessed their experience of wearing an Empatica E4 wristband and a sock-integrated Shimmer device, including their ease of use and level of distraction caused by the device. Accordingly, the questionnaire can be divided into four constructs, each with seven items: *Satisfaction experiment, Satisfaction Robot*, *wearing comfort Empatica E4*, and *wearing comfort sock*. Sum scores for each subscale ranged between 7 and 35, with higher scores indicating a better experience. Cronbach's alpha coefficients ranged between .70  – .91, suggesting acceptable to high internal consistency.

### 1.6. Data processing

Data extraction and segmentation were done with MATLAB. The raw skin resistance data from the Shimmer device was converted to skin conductance data using the formula: . For both the Shimmer data and the Empatica E4 data, the mean and standard deviation of the SC for the entire session per participant were calculated. Next, the data was standardized by transforming each SC value into a Z-score using the formula: Z−score=(SCvalue−meansession)/SDsession. By session, we intend the full signal for the entire experimental session. The raw SC data and the standardized SC data were segmented into five exercises based on notes made by the test leader. We did not distinguish between the tonic (skin conductance levels; SCL) and phasic (skin conductance responses; SCR) electrodermal activity. Both SCL and SCRs reflect sympathetic nervous system activity and show sensitivity to arousal/stress. As such, we had no specific hypotheses over SCL or SCR. The data was visually inspected for normality and outliers. No substantial deviations nor extreme outliers (3SD above and below the mean) were identified for physiological and behavioral data. A plot of single-participant data illustrating the raw skin conductance signal derived from each of the two devices is presented in S1.

### 1.7. Statistical analyses

Data analyses were performed in SPSS. First, descriptive analyses were conducted to characterize the sample (Table 1). A Bland–Altman analysis and illustrations were performed in R. This analysis was used to determine the agreement between the standardized skin conductance measurements of the Empatica E4 device and the sock-integrated Shimmer device. For this analysis, the difference between devices was calculated by subtracting the sock-integrated Shimmer’s skin conductance measurement from Empatica E4’s skin conductance measurements. The limits of agreement were then calculated by subtracting (lower limit) or adding (upper limit) 2 times the SD to the mean of the difference between the devices. The minimal detectable change (MDC) was also calculated by subtracting the lower limit of agreement from the upper limit of agreement and dividing this outcome by 2. When both devices measure the same outcome, the MDC should equal or below a threshold value relevant to the measured outcome value. For this study, a threshold value of 5 mS was selected. Secondly, an intra-class correlation coefficient (ICC) analysis was used to determine the agreement between the Empatica E4 and the Shimmer devices. A two-way random effects model with a multiple rater type and a definition of absolute agreements model compared the standardized skin conductance measurement of both devices.

Furthermore, after testing the assumption of normality of the skin conductance data with the Shapiro-Wilks test, it emerged that none of the exercises or devices followed a normal distribution. Accordingly, we conducted a nonparametric test with the Mann–Whitney-Wilcoxon U-test to estimate mean differences across the two devices for each exercise. Next, we computed the average SC for each data segment (baseline + 4 exercises) and calculated the correlation coefficient between the signal of the Shimmer device and the Empatica band.

For our second aim, several repeated measures ANOVAs were conducted with exercise (4) as a within-subjects factor, separately for skin conductance level recorded by Empatica E4 and the Shimmer device, and self-report arousal and valence, based on SAM. Planned comparisons were tested between each stress-inducing exercise and the relaxation exercise separately for each device.

## 2. Results

### 2.1. Preliminary results

The sample contributing data to this study was balanced concerning sex assigned at birth (50% females). The age ranged between 18 and 30 years, and most had completed a higher education degree (71%). Overall, participants reported positive feelings concerning the social validity of the study and the devices employed. Demographic characteristics are summarized in Table 1.

**Figure 3.**
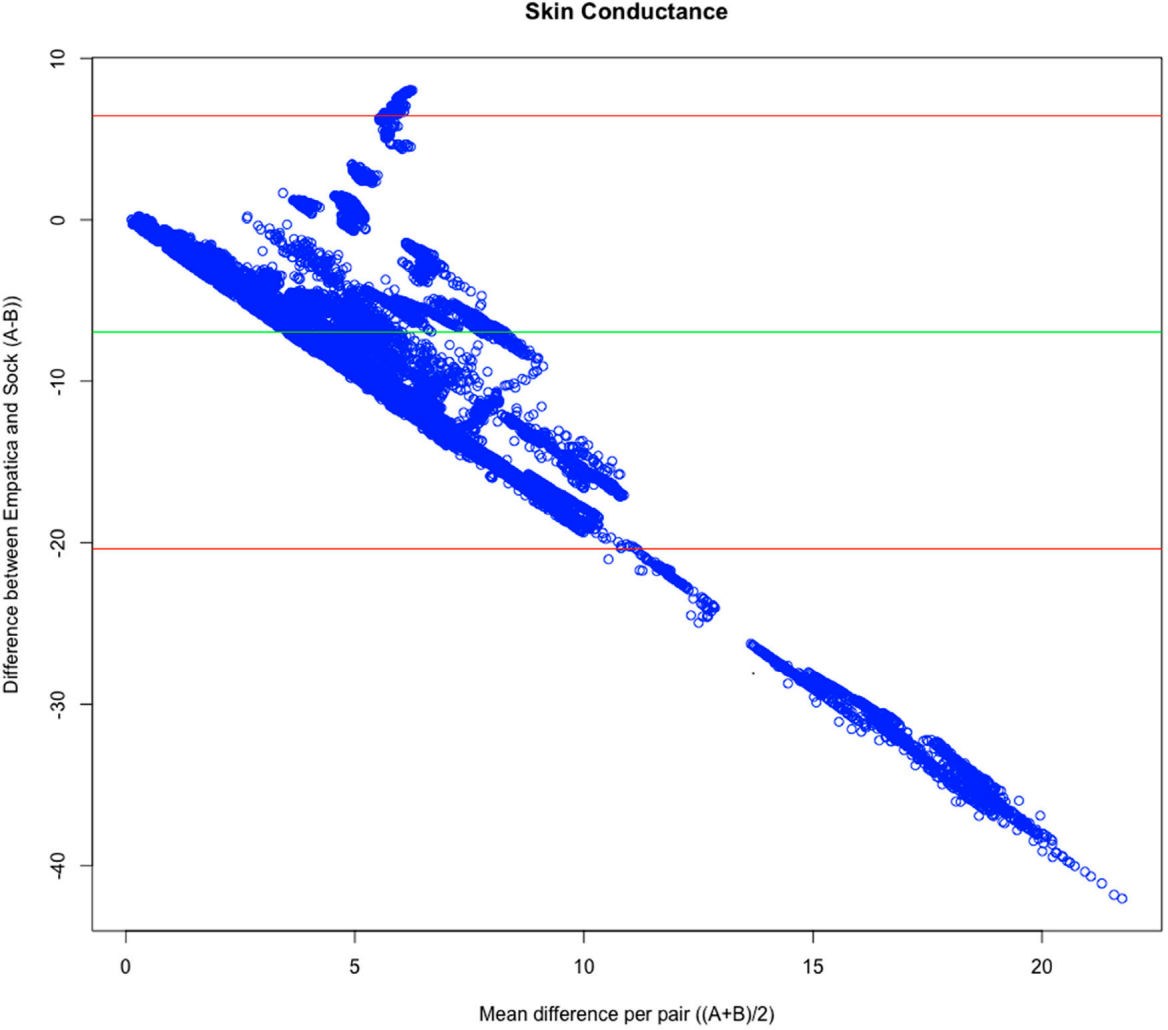
Bland-Altman plot for raw skin conductance indices of the sock-integrated Shimmer device and Empatica E4. The y-axis represents the difference between the two devices, and the x-axis represents the mean difference between the two devices.

The Bland–Altman analysis results, shown in Figure 3, indicate the level of agreement between the Shimmer and Empatica devices. The agreement range of – 0.165 to – 0.158 suggests discrepancies between the two devices. The minimal detectable change (MDC) of – 0.003 and the intraclass correlation coefficient (ICC) of 0.027 further indicate poor reliability in the data agreement. This suggests that the measurements from the two devices are inconsistent, implying that they may not be interchangeable for capturing electrodermal activity.

Table 2 presents the means and standard deviation for SC for each exercise and their differences and correlation coefficients between the two devices. The results show no statistically significant differences across the two devices during baseline, while the SC for the Shimmer was significantly higher than that for the Empatica E4 device. The SC for the Empatica E4 and the Shimmer were not significantly correlated, except for one inverse association for the first relaxation exercise. Within each device, the SC was highly correlated across all exercises.

**Table 2.** Pearson correlation coefficients between Empatica E4 and Shimmer device skin conductance across baseline and the four exercises.

	E4 *M (SD)*	S *M (SD)*	*U*-test	*r* (E4-S)	1.	2.	3.	4.	5.
1. Baseline exercise	.41 (.19)	.37 (.17)	.709	.021		.581**	.018	.039	.015
2. Stress exercise I	.40 (.16)	.57 (.18)	−3.617**	-.034	.833**		.848**	.686**	.495**
3. Relaxation exercise I	.37 (.15)	.57 (.19)	−3.650**	-.394*	.787**	.644**		.694**	.713**
4. Stress exercise II	.43 (.18)	.67 (.19)	−5.156**	.087	.542**	.599**	.725**		.628**
5. Relaxation exercise I	.35 (.15)	.58 (.17)	−4.987**	-.073	.592**	.586**	.790**	.79**	

Notes*.* E4 = Empatica E4 skin conductance level, S = Shimmer skin conductance signal, M = mean, SD = standard deviation, U = Mann-Whitney-Wilcoxon non parametric u-test, r = correlation coefficient. The blue shading highlights the correlation coefficient between SC for the same exercise. The green shading highlights the correlations across exercises measured with the Empatica E4. The yellow shading highlights the correlations across exercises measured with the Shimmer. *p < .05, **p < .01

### 2.2. Main results

#### 2.2.1. Wearing comfort

Participants’ ratings of wearing comfort of the Empatica E4 wrist-worn (*M *= 25.92, *SD *= 3.14) did not differ significantly *t*(27) = .106, *p *= .916 from that of the Shimmer device (*M *= 26, *SD *= 4.11).

#### 2.2.2. Physiological arousal following the Robot-delivered relaxation exercises

Results from the repeated measures ANOVA testing mean differences between the stress and relaxation exercises yield a significant difference in skin conductance recorded by the Empatica E4 device across exercises *F*(2.35, 25) = 3.897, *p *= .016. The sphericity assumption was violated (Mauchly's *W *= .522, *p *= .005); thus, we reported the Greenhouse-Geisser statistic. Planned comparisons with paired sample t-tests showed a statistically significant decrease in skin conductance between the second stress-eliciting exercise and the relaxation guided by the Robot through the visualization technique *t*(27) = 2.859, *p* = .008. For the first breathing exercise following the stress-eliciting math exercise, a decrease in arousal as indexed by skin conductance was found but did not reach statistical significance *t*(27) = 1.93, *p* = .064.

**Figure 4.**
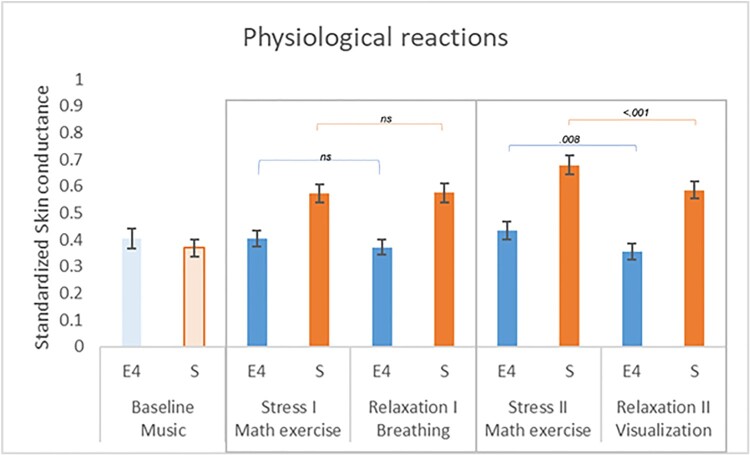
Illustration of the physiological reactions, indexed by skin conductance (y-axis), recorded by the Empatica E4 (E4, blue) and by the Shimmer device (S. orange) across the different exercises (x-axis). *ns *= non-significant, the error bars represent the standard deviations of the data.

The results were replicated when we tested mean differences in skin conductance as recorded through the Shimmer device *F*(3, 25) = 6.671, *p *< .001. The sphericity assumption was met. Planned comparisons with paired sample t-tests showed a statistically significant decrease in skin conductance between the second stress-eliciting exercise and the relaxation guided by the Robot through the visualization technique *t*(27) = 4.083, *p* < .001. For the first breathing exercise following the stress-eliciting math exercise, no statistically significant differences emerge *t*(27) = -.101, *p* = .920. Results are illustrated in Figure 4. The baseline condition was displayed for illustrative purposes as a sanity check that participants were relaxed before the start of the first stress exercise.

### 2.3. Behavioral arousal and valence following the Robot-delivered relaxation exercises

Results from the repeated measures ANOVA testing mean differences between the stress and relaxation exercises yield a significant difference in self-reported arousal across exercises *F*(1.093, 25) = 41.067, *p *< .001. The sphericity assumption was violated (Mauchly's *W *= .012, *p *< .001); thus, we reported the Greenhouse-Geisser statistic. Planned comparisons with paired sample t-tests showed a statistically significant decrease in self-reported arousal between the first stress-eliciting exercise and the relaxation guided by the Robot through the breathing technique *t*(27) = 4.322, *p* < .001. For the second exercise, using the visualization technique following the stress-eliciting math exercise, results showed a statistically significant decrease in self-reported arousal *t*(27) = 6.502, *p* < .001.

**Figure 5.**
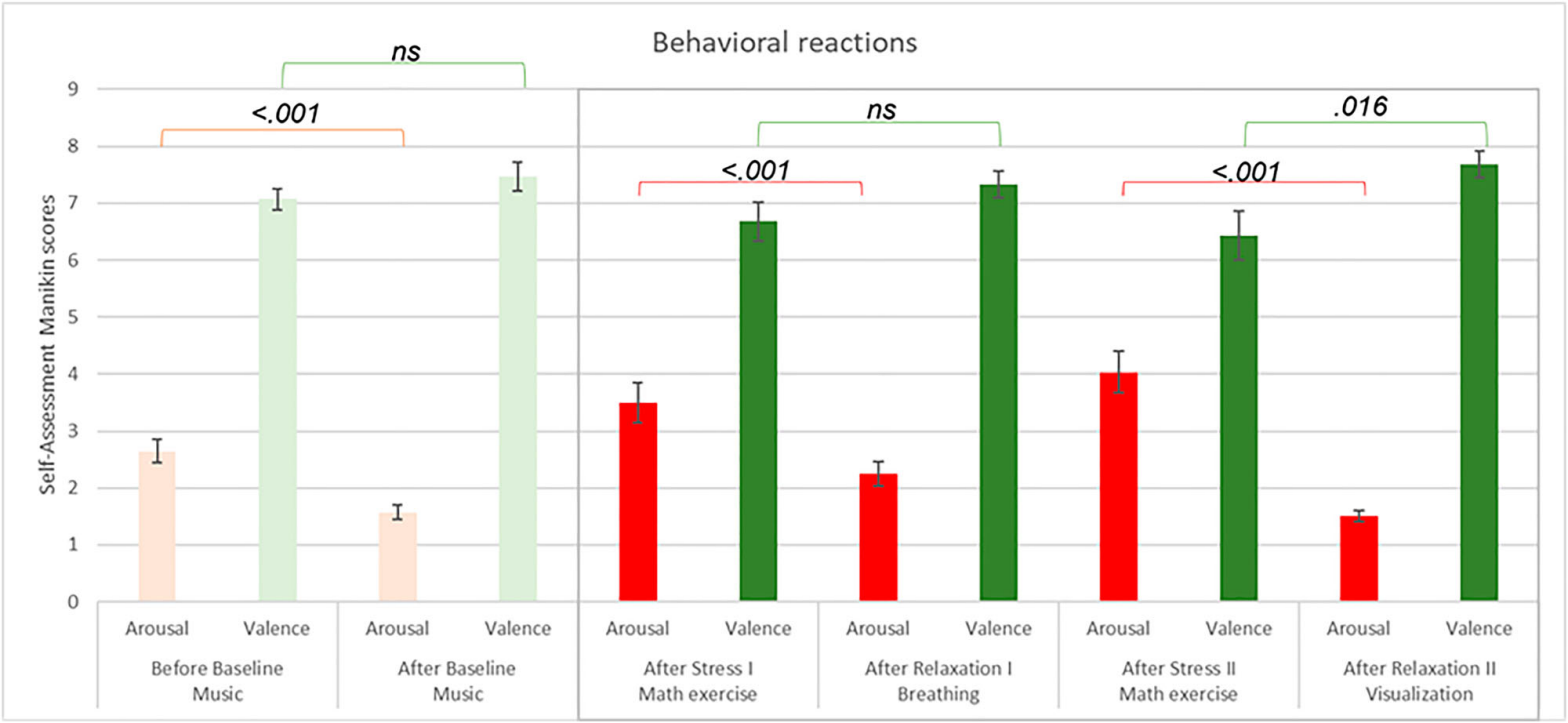
Illustration of the behavioral reactions, indexed by arousal (red) and valence (green) (y-axis), self-reported with the Self-Assessment Manikin across the different exercises (x-axis). *ns *= non significant, the error bars are the standard deviations of the data.

The results were replicated when we tested mean differences in valence *F*(1.05, 25) = 6.175, *p *= .018. The sphericity assumption was violated (Mauchly's *W *= .004, *p *< .001); thus, we reported the Greenhouse-Geisser statistic. Planned comparisons with paired sample t-tests showed a statistically significant increase in positive valence between the second stress-eliciting exercise and the relaxation guided by the Robot through the visualization technique *t*(27) = 2.582, *p* = .016. For the first breathing exercise following the stress-eliciting math exercise, an increase in positive valence was found but did not reach statistical significance *t*(27) = 1.782, *p* = .086. Results are illustrated in Figure 5. The baseline condition was displayed for illustrative purposes as a sanity check that participants were relaxed before the start of the first stress exercise.

## 3. Discussion

The current study studied the sensitivity of two wearable devices recording physiological arousal or electrodermal activity, indexed by skin conductance and recorded via the wrist-worn Empatica E4 wristband and the Shimmer device connected to a sock wearable. Physiological recordings were recorded during a humanoid Robot-delivered mindfulness-based intervention to reduce stress. We found that both the Empatica E4 and the Shimmer device showed sensitivity in detecting variations in skin conductance as a function of stress exercises and the Robot-delivered relaxation exercises. Self-reports of arousal and valence substantiated these physiological results. Participants did not report differences in wearing comfort between the Empatica E4 and the Shimmer. However, poor correspondence was found between the two devices.

Our first hypothesis regarding the correspondence between the two systems was only partially supported by our findings. We expected no differences between the Shimmer device and the wrist-worn Empatica E4 band regarding sensitivity to capture skin conductance signals in response to the Robot-delivered manipulations. The two devices have independently found an increase in skin conductance following a stressor and a decrease following a relaxation exercise. Interestingly, when directly comparing the signals of the two devices, we found no significant relation between the skin conductance signals of the two recording devices. Besides differences in the recording device, the different anatomical locations might have played a role in these findings [44]. Similar findings have been described in a recent study comparing the Empatica E4 wrist-worn and a foot-located Shimmer [45]. Another difference worth considering is the sampling frequency difference (50 Hz for Shimmer^TM^ and 4 Hz for Empatica E4). In this study, we aligned the measures using standardization to reduce non-experimentally manipulated intra  – and inter-individual differences [46]. During one exercise, we found a significant negative relation between the two types of devices. This puzzling finding has also been reported in an earlier study [45], suggesting that bodily reactions may not converge in the same direction from all anatomical standpoints. An increase in the sweat glands may not linearly follow the same activation pattern across all sites. This could be partly explained from a multiple arousal theory standpoint, suggesting that the autonomic nervous system may reflect differential electrodermal activity across sites and body laterality due to hemispheric asymmetry to emotional responses [47,48]. Differences in the laterality, recording site, and devices may contribute to the lack of a significant relation between the recorded signals. Furthermore, we tested for linear relations using SC. Still, it could be the case that patterns of EDA from different sites do not reflect a linear relation, and different markers of the skin conductance should be analyzed (i.e. latency, frequencies, peak-to-peak). We did not assess laterality systematically or record the body side. Given the goal of our study to assess sensitivity to experimental manipulation of the two devices independently, such analyses remain outside the scope of this paper but provide intriguing future questions to address further.

In our study, we employed both devices simultaneously during a stress-reducing Robot-delivered intervention. Considering the sensitivity of the Shimmer and the Empatica E4 to detect changes in physiological stress as a function of stress and relaxation conditions, we found that both systems yielded similar results. Participants showed decreased physiological arousal during the Robot-delivered mindfulness-based exercises, with significant effects on the visualization exercise in both device recordings. The results suggest that the skin conductance signals from the Empatica E4 and the Shimmer device connected to a sock were independently registered accurately across manipulation of stress and relaxation. Upon closer inspection of Figure 4 depicting the baseline electrodermal activity, it seems that the magnitude of variation recorded by the Empatica E4 device is lower than that recorded by the Shimmer device. This may be due to different amplifiers integrated into the technology or physiological and anatomical differences in recording sites. However, it could also be considered that the Empatica E4 may be less sensitive in recording subtle variations in electrodermal activity, especially when not strongly arousing stimulation. This may be explained by the lower capacity of dry electrodes integrated into wrist-worn devices because they need a higher amount of sweat to detect EDA [23]. Future studies employing multiple recording anatomical sites and larger samples should confirm these possible explanations. Furthermore, participants reported similar preferences for both from a comfort perspective of wearing the device. Accordingly, it can be concluded that any of the two devices can be reliably employed in this type of research. Moreover, future studies should also include a subjective measure of anxiety to control for possible white-coat effects [49,50], which may produce higher cardiac activity in some participants.

The Robot-delivered stress reduction intervention, rooted in Mindfulness practices [51,52], has shown positive effects both on a physiological level, as described above, and on a behavioral level. Participants reported reductions in arousal from the stress-eliciting math exercises in response to both exercises (breathing and visualization), although only the visualization exercise reached statistical significance. Likewise, participants reported an overall increase in self-reported pleasure (valence) following the relaxation exercises. These findings match the physiological data, providing comprehensive evidence of the effectiveness of an NAO Robot-delivered intervention to reduce stress. While our results align with literature in typically developed individuals [2,8], they contrast behavioral findings in individuals with intellectual disabilities [10]. In the study by Van Wingerden [10], individuals with ID did not report statistically significant reductions in arousal following the Robot-delivered relaxation exercises. Interestingly, qualitative data indicated that clients may have benefitted from the intervention, leaving a gap in our understanding of such mixed findings. One possible explanation is that clients with cognitive impairments may have difficulty correctly reporting their affective states. To this end, integrating behavioral data and physiological markers of arousal or stress becomes imperative. In conclusion, this study provides comprehensive insights into the effectiveness of the intervention from a psychophysiological perspective.

These findings provide evidence of different devices’ sensitivity in assessing skin conductance in response to stress and relaxation conditions. The physiological evidence paralleled psychological self-reports, strengthening our conclusions. Additionally, both devices have been rated as equally comfortable to wear. This evidence offers new venues for research in the field of disability or possibly pediatric populations, in which self-report measures may not be appropriate in capturing implicit subtle changes in affective states. Considering the choice of a Shimmer device or Empatica E4, while both devices have been deemed valid, they can be used considering the participants and the experiment. For example, 1) when working with people with severe or profound intellectual disabilities, the sock-integrated Shimmer device is potentially more acceptable since they may have more experience with wearing socks daily but do not bracelets or watches, which may be more distracting; 2) in an experiment in which participants were asked to wiggle their toes, the Empatica E4 would be a better choice above the sock-integrated Shimmer as the data would then be prone to movement artifacts. Accordingly, researchers may inform their choice of a device, depending on the exercises they plan to use, to limit motion artifacts and sensor disconnection from the anatomical site.

While our study provides valuable insights, it also has some limitations that warrant caution in interpreting our findings and call for future research to substantiate them. First, we employed the skin conductance measure as the average of the signal during a specified time window without differentiating between the tonic and phasic domains of electrodermal activity. While we aimed to simply test variations in two independent devices as a function of experimental manipulation embedded in Robot-delivered stress and relaxation exercises, more sophisticated analyses of the skin conductance level versus response, as well as temporal dynamics of change and frequency, may provide invaluable insights into the accuracy of these recording devices. In this study, the order of the exercises was fixed. Thus, it remains unclear whether the difference between the second stress-inducing and relaxation exercise is due to the order or because the visualization exercises may be more effective in stress recovery. Future studies should further address the type of exercises used in such interventions. Another limitation concerns the laterality of the recording. Unfortunately, we did not log which body side was used, and both devices were placed on the same side, preventing us from clarifying possible differences as claimed by the multiple arousal theory. Last, given the small sample size, future studies with larger samples are needed to confirm these preliminary results. These limitations underscore the need for further research in this area, and we encourage our colleagues to continue this important work.

This foundational study provides preliminary evidence for integrating wearables for physiological recordings during a well-established therapeutic practice delivered by a humanoid Robot. Further, our findings revealed equally sensitive and comfortable skin-conductance recording devices (Shimmer device and wrist-worn Empatica E4).

## Data Availability

Data is available on request from the corresponding author.
